# Exploring Behavioral Patterns for Data-Driven Modeling of Learners' Individual Differences

**DOI:** 10.3389/frai.2022.807320

**Published:** 2022-02-15

**Authors:** Kamil Akhuseyinoglu, Peter Brusilovsky

**Affiliations:** School of Computing and Information, University of Pittsburgh, Pittsburgh, PA, United States

**Keywords:** individual differences, learner modeling, sequential pattern mining, learning technology, online practice, SQL

## Abstract

Educational data mining research has demonstrated that the large volume of learning data collected by modern e-learning systems could be used to recognize student behavior patterns and group students into cohorts with similar behavior. However, few attempts have been done to connect and compare behavioral patterns with known dimensions of individual differences. To what extent learner behavior is defined by known individual differences? Which of them could be a better predictor of learner engagement and performance? Could we use behavior patterns to build a data-driven model of individual differences that could be more useful for predicting critical outcomes of the learning process than traditional models? Our paper attempts to answer these questions using a large volume of learner data collected in an online practice system. We apply a sequential pattern mining approach to build individual models of learner practice behavior and reveal latent student subgroups that exhibit considerably different practice behavior. Using these models we explored the connections between learner behavior and both, the incoming and outgoing parameters of the learning process. Among incoming parameters we examined traditionally collected individual differences such as self-esteem, gender, and knowledge monitoring skills. We also attempted to bridge the gap between cluster-based behavior pattern models and traditional scale-based models of individual differences by quantifying learner behavior on a latent data-driven scale. Our research shows that this data-driven model of individual differences performs significantly better than traditional models of individual differences in predicting important parameters of the learning process, such as performance and engagement.

## 1. Introduction

Learners' individual differences have been recognized as a critical factor in the learning process. A wide range of cognitive, personal, motivational, and other dimensions of individual differences was introduced by researchers in the areas of cognitive science and educational psychology (Jonassen and Grabowski, 1993). However, traditional dimensions of individual differences haven't yet proven their value in addressing the needs of modern e-learning. In particular, there are few successes in using these differences in solving the important problem of predicting student performance and engagement (Upton and Adams, 2006; Chen et al., 2016; An and Carr, 2017). At the same time, e-learning research has demonstrated that the large volume of learning data collected by modern e-learning systems could be used to recognize student behavior patterns and connect these patterns with measures of student performance (Sharma et al., 2015; Geigle and Zhai, 2017; Hansen et al., 2017; Boroujeni and Dillenbourg, 2018; Lorenzen et al., 2018; Mouri et al., 2018; Gitinabard et al., 2019).

In this paper, we attempt to bridge these research directions by developing a data-driven model to capture individual differences that were possibly exposed through practice behavior in an e-learning system and by representing learner behavior on a continuous scale following the traditional work on modeling individual differences. After reviewing related literature (Section 2) and introducing the online practice system, datasets, measures and procedures used in this study (Section 3), we start our analysis by examining connections between the individual differences measured at the start of the learning process (“*incoming” parameters*) with learner engagement and performance (Section 4). Next, we build a simple model of learner practice behavior during the usage of the online system. We achieve this goal in two steps. On the first step, we build representative profiles of individual practice behavior by applying sequential pattern mining to the logs of an online practice system (Section 5). On the second step, we cluster individual profiles to reveal student subgroups that demonstrate noticeably different practice behavior (Section 6). The obtained clusters represent a simple formal model of learner practice behavior where individual students are modeled by association with one or another cluster. We use this model for our first examinations of connections between learner behavior and other parameters of the learning process such as individual differences and performance (Section 7). The obtained results indicated that our simple cluster model captures important individual differences between learners not represented by the traditional dimensions of individual differences used in our study. Following this encouraging results, we proceed with refining the simple cluster model into a complete data-driven model of individual differences in a form of a continuous behavior scale (Section 8), similar to traditional models of individual differences, such as the achievement goal orientation framework and the self-esteem scale (Rosenberg, 1965; Elliot and McGregor, 2001). We assess the value of the data-driven model by comparing the predictive power of this model to the traditional models of individual differences. Our results indicate that the data-driven model performs significantly better than traditional models of individual differences in predicting learner performance and engagement. Further, we demonstrate the transferability of our model by evaluating its predictive power on a new dataset.

## 2. Related Work

### 2.1. Individual Differences and Academic Achievement

Individual differences have been the focus of research on educational psychology and learning technology (Jonassen and Grabowski, 1993). Numerous works have attempted to discover and examine various *dimensions* of individual differences, find their connections to academic achievement, and address these differences in order to better support teaching and learning. A learner's position within a specific dimension of individual differences is usually determined by processing carefully calibrated questionnaires and placing the learner on a linear scale, frequently between two extreme ends. In this section, we briefly review several dimensions of individual differences that are frequently used in learning technology research.

*Self-efficacy* refers to one's evaluation of their ability to perform a future task (Bandura, 1982) and is shown to be a good predictor of educational performance (Multon et al., 1991; Britner and Pajares, 2006). Students with higher self-efficacy beliefs are more willing to put effort into learning tasks and persist more, as compared to students with lower self-efficacy. *Self-esteem* represents individuals' beliefs about their self-worth and competence (Matthews et al., 2003). Some studies have shown the positive effect of self-esteem on academic achievement, while other studies have pointed out how academic achievement affects self-esteem (Baumeister et al., 2003; Di Giunta et al., 2013). Researchers also stated the indirect effect of low self-esteem on achievement through distress and decreased motivation (Liu et al., 1992). Learners can also differ by their *achievement goals*, which guide their learning behaviors and performance by defining the expectations used to evaluate success (Linnenbrink and Pintrich, 2001). Studies have demonstrated the positive effects of achievement goals on performance (Harackiewicz et al., 2002; Linnenbrink and Pintrich, 2002). There are several known questionnaire-based instruments to capture achievement goals (Midgley et al., 1998; Elliot and McGregor, 2001).

Another important group of individual differences is related to metacognition, which plays an important role in academic performance (Dunning et al., 2003). In particular, students who successfully distinguish what they know and do not know can expand their knowledge instead of concentrating on already mastered concepts (Tobias and Everson, 2009). It has been shown that high-achieving students are more accurate in assessing their knowledge (DiFrancesca et al., 2016). To measure some metacognitive differences, Tobias and Everson (Tobias and Everson, 1996) proposed a *knowledge monitoring assessment* instrument to evaluate the discrepancy between the actual performance of students and their own estimates of their knowledge in a specific domain.

### 2.2. User Behavior Modeling and Performance Prediction

The rise of interest to modeling learner behavior in online learning system is associated with attempts to understand learner behavior in early Massive Open Online Courses (MOOCs) with their surprisingly high dropout rate. Since MOOCs usually recorded full traces of learner behavior producing rich data for a large number of students, it was natural to explore this data to predict dropouts (Balakrishnan, 2013) and performance (Anderson et al., 2014; Champaign et al., 2014). This appealing research direction quickly engaged researchers from the educational datamining community who were working on log mining and performance prediction in other educational contexts and led to a rapid expansion of research that connected learner behavior with learning outcomes in MOOCs and beyond.

While the first generation of this research focused on one-step MOOC performance prediction from learning data (Anderson et al., 2014; Champaign et al., 2014; Boyer and Veeramachaneni, 2015; Brinton and Chiang, 2015), the second generation attempted to uncover the roots of performance differences to better understand the process and improve predictions. The core assumption of this stream of work was the presence of latent learner cohorts composed of students who exhibit similar patterns. By examining connections between these cohorts and learning outcomes, the researchers expected to identify positive and negative patterns and advance from simple prediction of learner behavior to possible interventions. While the idea of cohorts was pioneered by the first generation research, the early work on cohorts attempted to define them using either learner demographic (Guo and Reinecke, 2014) or simple activity measures (Anderson et al., 2014; Sharma et al., 2015). In contrast, the second generation research attempted to automatically discover these cohorts from available data. Over just a few years, a range of approaches to discover behavior patterns and use them to cluster learners into similarly-behaving cohorts were explored. This included various combinations of simple behavior clustering (Hosseini et al., 2017; Boubekki et al., 2018), transition analysis (Boubekki et al., 2018; Gitinabard et al., 2019), Markov models (Sharma et al., 2015; Geigle and Zhai, 2017; Hansen et al., 2017), matrix factorization (Lorenzen et al., 2018; Mouri et al., 2018; Mirzaei et al., 2020), tensor factorization (Wen et al., 2019), sequence mining (Hansen et al., 2017; Hosseini et al., 2017; Venant et al., 2017; Boroujeni and Dillenbourg, 2018; Mirzaei et al., 2020), random forests (Pinto et al., 2021), and deep learning (Loginova and Benoit, 2021). In this paper, we focus on the sequence mining approach to behavior modeling which is reviewed in more detail in the next section.

### 2.3. Sequential Pattern Mining

In educational research, mining sequential patterns has become one of the common techniques to analyze and model students' activity sequences. This technique helped researchers to find student learning behaviors in different learning environments. Nesbit et al. (2007) applied this technique to find self-regulated behaviors in a multimedia learning environment. In Maldonado et al. (2011), authors identified the most frequent usage interactions to detect high/low performing students in collaborative learning activities. To find differences among predefined groups (e.g., high-performing/low-performing), Kinnebrew and Biswas (2012) proposed a differential sequence mining procedure by analyzing the students' frequent patterns. Herold et al. (2013) used sequential pattern mining to predict course performance, based on sequences of handwritten tasks. Guerra et al. (2014) examined the students' problem solving patterns to detect stable and distinguishable student behaviors. In addition, Hosseini et al. (2017) used a similar approach to Guerra et al. (2014) and detected different student coding behaviors on mandatory programming assignments, as well as their impact on student performance. Venant et al. (2017) discovered frequent sequential patterns of students' learning actions in a laboratory environment and identified learning strategies that associated with learners' performance. Mirzaei et al. (2020) explored specific patterns in learner behavior by applying both sequential pattern mining and matrix factorization approaches. In the earlier version of this paper (Akhuseyinoglu and Brusilovsky, 2021), the authors applied the sequence mining approach to analyzing student behavior in an online practice system.

## 3. Methods

**Figure 1:**
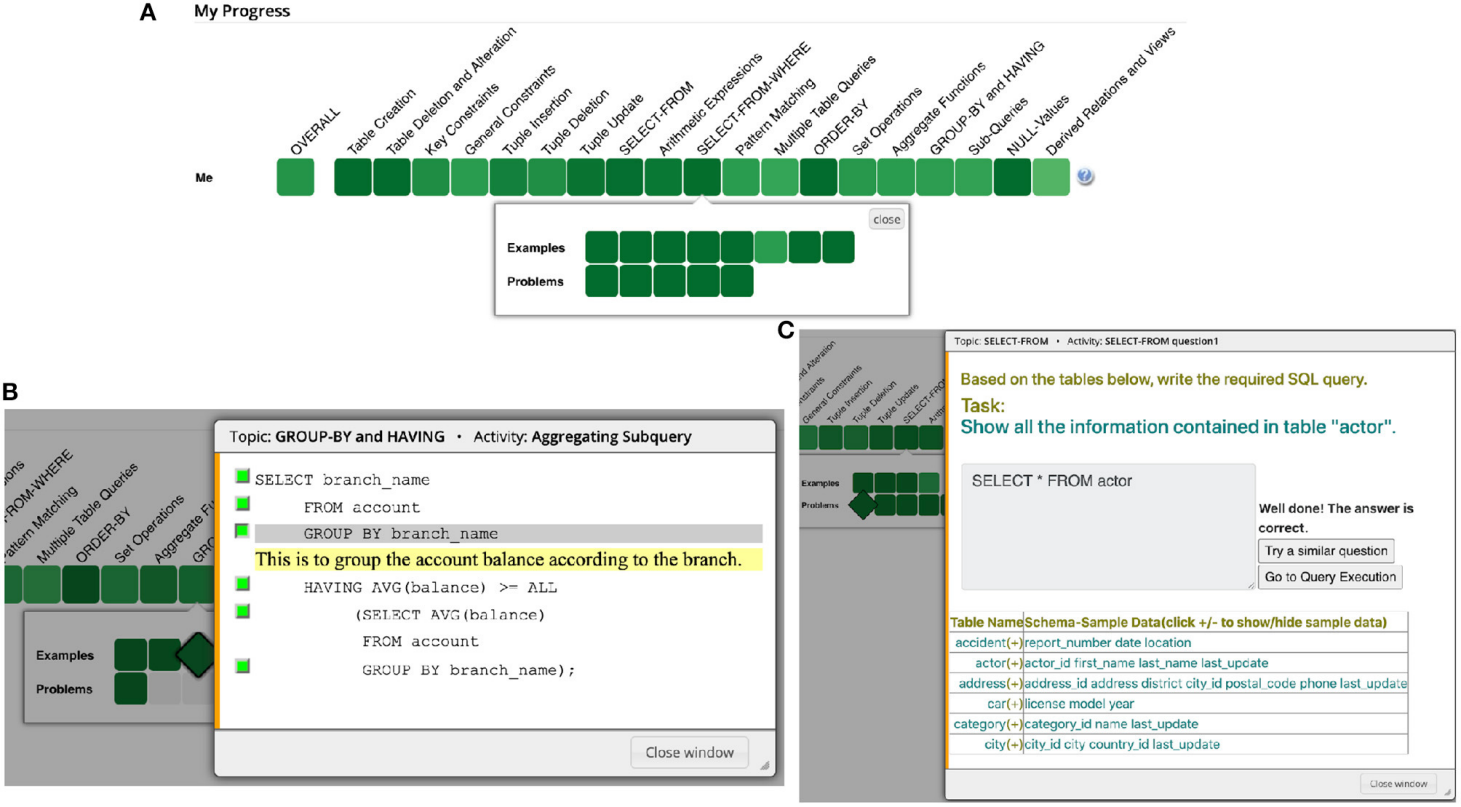
**(A)** SQL practice system offered access to SQL examples and problems organized by topics. The figure shows the list of available learning content for the topic “SELECT-FROM-WHERE” **(B)** An instance of an example in the “group-by and having” topic in the SQL practice system. Here, the student has clicked on the third line and an explanation is shown below the line that demonstrates the usage of group by clause. **(C)** An instance of a problem in the “select-from” topic in the SQL practice system. Here, the student successfully solved the problem and system provided an immediate feedback. Next, the student can try solving a similar problem or switch to query execution mode to see the actual query results.

**Figure 2:**
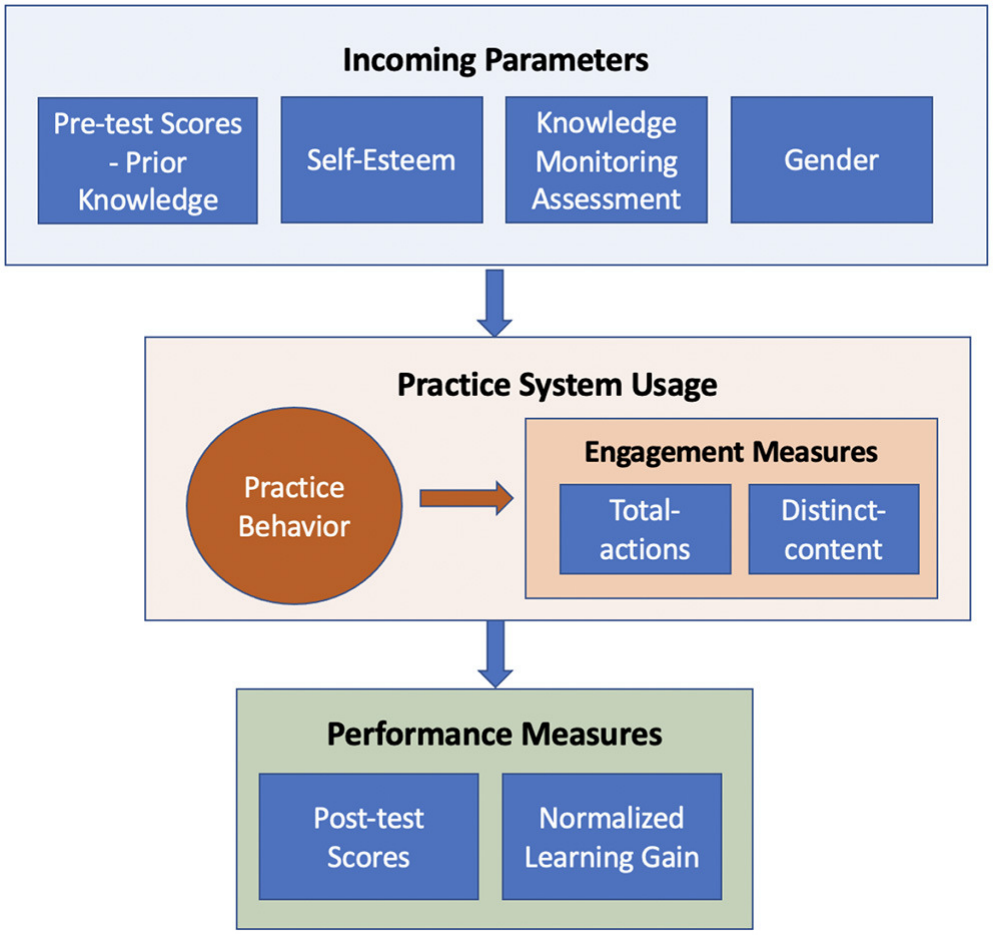
An overview of key measures used in the study. We explored possible connections between learners' individual differences (i.e., incoming parameters) collected at the start of the learning process, practice behavior, performance and engagement measures. We used practice system usage logs to model practice behavior of learners and constructed a data-driven model of individual differences.

In this paper, we explore connections (**Figure 2**) between incoming parameters of the learning process (i.e., individual differences, prior knowledge, gender), learner practice behavior, and outgoing parameters (engagement and performance) by examining activity logs of an online practice system for SQL programming (Figure 1). The system was available over several semesters to students taking a database class. The non-mandatory nature of the system allowed students to decide when and how much to practice and increased their chances to expose individual differences through their free practice behavior. This section explains in detail the nature of the practice system, components of the dataset. In addition, we defined the important measures and procedures used in performance and engagement prediction.

### 3.1. The Course and the Online Practice System

In this study, we use data collected from four semesters of classroom studies in a graduate level Database Management course at a large North American university. Learning Structured Query Language (SQL) was one of the objectives of the course. The structure of the course remained the same for all four semesters, including the syllabus and the grading policy.

The SQL practice system (Brusilovsky et al., 2016) was offered to all classes as a non-mandatory tool for learning and self-assessment. The system provided access to two types of interactive learning content: SQL problems focused on SQL SELECT statements and annotated examples of SQL statements (Brusilovsky et al., 2010). The content was grouped into topics, and each topic had multiple problems and examples (Figure 1A). Students could freely choose the topic and the content to practice. To encourage the students to explore the practice system, one percentage point of extra credit was provided to the students who solved at least 10 SQL problems.

The worked-out examples (Figure 1B) offered complete examples of SQL code augmented with explanations, which students could examine interactively, line by line. The *problems* were designed to help students practice their SQL code writing skills. Each problem was *parameterized*; i.e., generated from a set of pre-defined templates with randomly selected parameters (Figure 1C). This design allowed students to practice the same problem multiple times. The correctness of their responses was tested against a fixed database schema and immediate correct/incorrect feedback was provided (Figure 1C). The problem tool also offered a *query execution mode*, which allows students to execute their SQL queries while working with the problems and see the actual query results in the form of tables. This tool allowed students to check their SQL code before submitting it as an answer to a problem. Altogether, the system allowed students to study 64 annotated examples with 268 distinct explained code lines and to practice code writing with 46 problem templates.

### 3.2. The Dataset Collection

The collected dataset included the activity logs of the online practice system, knowledge measures, such as pre/post test scores, and several questionnaires focused on identifying learners' individual differences. Below, we explained collected metrics in more detail.

**Knowledge Measures:** To measure overall knowledge improvement throughout the course, a *pre-test* and a *post-test* were administered. Before the SQL topics were introduced, a pretest was administered. At the end of the semester, a post-test was administered. Both tests consist of 10 problems covering data definition, data query and data manipulation SQL statements related to a given database schema. Post-test problems were isomorphic to the pretest. The *normalized learning gain* (NLG) was calculated as the ratio of the actual gain to the maximum possible gain as follows:


(1) $$\begin{array}{l} \text{NLG}=\left(\text{post}-\text{pre}\right)/\left(\text{max}\text{\_}\text{possible}\text{\_}\text{post}-\text{pre}\right) \end{array}$$

Each test had 10 problems that required writing SQL statements. Reported pre- and post-test scores ranged between 0 and 10.

**Questionnaires:** We collected gender data, and used several instruments to measure individual differences. To measure *global self-worth*, in the first three semesters we administered a 10-item Rosenberg Self-Esteem Inventory (Rosenberg, 1965). Responses (α = 0.82) were converted to a continuous scale (ranges from 0 to 30) where higher scores indicate higher *self-esteem* (SE). To perform a *Tobias-Everson knowledge monitoring assessment* (KMA) (Tobias and Everson, 1996), we asked students to estimate correctness of their answers to 10 pre-test problems. This way, we evaluated the differences between a students' actual performance and her confidence for each problem. A correct estimate means that a student answered a problem correctly/incorrectly and estimated that she solved that problem correctly/incorrectly. Then, we counted the number of correct and incorrect estimates, and a score was computed as follows:


(2) $$\begin{array}{l} \text{KMA}=\left(\text{correct}\text{\_}\text{estimates}-\text{incorrect}\text{\_}\text{estimates}\right)/\text{total}\text{\_}\text{estimates} \end{array}$$

The KMA score ranges from −1 to 1, where a score of 1 indicates that the student knows perfectly what they know or do not know. In KMA score calculation, we only considered problems with an estimate.

**Activity Logs:** We collected students' timed interaction logs with the online practice system for each of the four consecutive semesters. The logs offer a detailed view of student practice behavior, including problem solving attempts, example and explanation line views, and query executions in the *query execution mode*.

We examined the differences in knowledge measures for the first three semesters and found out that there were no significant differences in pre-test scores [Kruskal-Wallis $\chi_{\left(2\right)}^{2}=5.03,p=0.08$], post-test scores [Kruskal-Wallis $\chi_{\left(2\right)}^{2}=1.10,p=0.58$], and NLG [*F*_(2, 78)_ = 1.13, *p* = 0.33]. These findings suggested that students cohorts were similar based on the knowledge measures. Given that we had the full set of individual difference data for the first three semesters, we combined the data from these semesters into the *main dataset*. The main dataset was used for building and evaluating the data-driven model of individual differences. Data collected during the fourth semester was used to test the transferability of the proposed model (Section 8.4) and we refer it as the *transfer dataset*. None of the students in the datasets repeated the course. Also, note that some students didn't respond to some of the instruments and pre/post tests. Table 1 presents the summary about collected metrics for each dataset. In this table and the remaining analysis, we only used the data collected from students who gave their consent and who tried the practice system by attempting at least one SQL problem and viewing at least one example. Detailed filtering process shared along with the reported analyses. For practice system usage, we reported the average number of attempted distinct problems, viewed distinct examples, and explanation lines, as well as the average number of query executions.

**Table 1:** Summary statistics of the main and transfer datasets.

		**Incoming parameters**				**Performance**		**Practice system usage**			
		**Female**	**SE**	**KMA**	**Pre-test**	**Post-test**		**Problem**	**Example**	**Line**	**Query**
**Dataset**	**N**	**%**	**score**	**score**	**score**	**score**	**NLG**	**attempts**	**views**	**views**	**executions**
Main	88	53	21.3(4.2)	0.54(0.43)	1.3(1.5)	4.9(1.7)	0.41(0.17)	33.2(16.4)	53.0(16.7)	122.6(70.3)	54.2(59.4)
Transfer	36	NA	NA	NA	1.9(1.9)	5.2(2.2)	0.40(0.24)	33.0(17.4)	51.2(19.0)	132.6(65.5)	57.8(75.6)

*Mean and (SD) are reported*.

### 3.3. Key Variables in the Study

In this section, we summarized the key parameters and measures used in the study. Figure 2 provides an overview of these variables and their connections.

**Incoming Parameters:** Throughout the paper, we used the term *incoming parameters* to refer to variables representing differences among students at the start of the course. These parameters include starting level of knowledge measured by the pre-test, gender, and traditional scales of individual differences such as SE and KMA scores. In the main dataset, SE and KMA scores were not correlated with each other and with the knowledge measures^1^.

**Performance Measures:** Performance represents the outcomes of the learning process and is the most typical measure of the learning process. To measure student *performance*, we used (1) post-test scores and (2) NLG as the objective performance measures collected outside the practice system.

**Engagement Measures:** Engagement is a less traditional group of metrics, yet it remains essential in the context of non-mandatory learner-driven practice. While practicing with interactive learning content is usually beneficial for the growth of learner knowledge, many students tend to ignore the opportunity to practice or practice very little. In this context, the engagement with practice becomes a critical factor of the learning process. Connecting individual differences to engagement measures is important to plan interventions and to improve learner performance.

Learner engagement measures have been extensively discussed in the research literature (Appleton et al., 2006; Grier-Reed et al., 2012). In modern e-learning, engagement is frequently approximated by the amount of student voluntary work (i.e., work not directly required and graded). For example, in MOOCs, engagement is frequently assessed by the fraction of watched videos, the number of attempted quizzes, or the number of posts to a discussion forum (Anderson et al., 2014; Davis et al., 2017; Crues et al., 2018). Similarly, online practice systems generally measure learner engagement through the amount of voluntary practice with worked examples and problems (Denny et al., 2018; Hosseini et al., 2020). Following this trend, we approximated *engagement* as the amount of students' non-mandatory work with different learning activities available in the practice system. The measures that we used for engagement are: (1) the total number of learning actions performed: calculated by summing up the number of problem solving attempts (regardless of correctness), the number of query execution attempts, the number of annotated examples viewed, and the number of line explanations viewed (referred as *total-actions*); and (2) the total number of distinct learning activities performed: calculated by summing up the number of distinct problems attempted (DPA), distinct annotated examples viewed (DEV), and distinct explanation lines viewed (DLV) (referred as *distinct-content*). Note that the *total-actions* measure counts duplicate accesses to the same learning content, such as opening the same example or attempting to solve the same problem more than once. Thus, this measure reflects the overall levels of engagement with the practice system. On the other hand, *distinct-content* incorporates uniqueness of the learning content and reflects overall content coverage by a student.

**Outgoing Parameters:** In our study, performance and engagement are considered to be *outgoing parameters* of the learning process because both could be affected by incoming parameters and learning behavior. However, the roles of these variables are not symmetric. While performance represents the final learning outcomes of the process, the engagement is a process variable that can impact the learning outcomes (Figure 2).

### 3.4. Engagement and Performance Prediction Procedure

Throughout the paper, we fitted multiple regression models to separately predict each performance and engagement measure and compared the overall fit of the models using likelihood ratio tests. In some cases, we compared simple regression models by checking the adjusted *R*^2^. Moreover, we compared the relative importance of features based on the regression estimates. We used negative binomial generalized linear models to predict count outcome variables due to over-dispersion. For other measures, we fitted simple linear regressions. We considered adding a random effect to the regression models to account for the variability in semesters, but given the very low estimated variance of the random effect, we continued with only the fixed effects models. In addition, we checked regression assumptions, including the multicollinearity, by calculating the *variance inflation factors (VIF)* and made sure that none of the features had $\sqrt{VIF}>2$.

For both prediction analyses, we used students' data from the main dataset who filled out the questionnaire, took the pre-test, attempted at least one problem, and viewed at least one example in the practice system. For engagement prediction, we used 70 students' data. For performance prediction analyses, we further excluded four students who did not take the post-test and who had zero NLG and used data of 66 students.

In performance prediction, we wanted to control for the students' “amount of practice” by adding user engagement measures to our regression models as covariates. To do so, we considered the total number of distinct problems attempted (DPA), distinct examples viewed (DEV), and distinct explanation lines viewed (DLV) as possible covariates. We performed a backward step-wise feature selection process and found out that *DPA* was the only feature that significantly predicts the post-test scores (after controlling by the pre-test scores) and NLG. Thus, we added DPA as a covariate to all regression models that fitted for performance prediction. To predict post-test scores, in addition to the DPA, we added pre-test scores to control for the levels of prior knowledge. However, for NLG prediction, we did not include pre-test scores as a feature, since it was already used to calculate NLG itself.

## 4. Using Incoming Parameters for Engagement and Performance Prediction

As explained above, one of the key goals of this paper is to explore connections between incoming parameters of the learning process, learner practice behavior, and outgoing parameters of the process (Figure 2). This goal can't be achieved directly using the data in our dataset because exploring connections of incoming and outgoing parameters with learning behavior requires building a model of this behavior. Building of this model requires a relatively complex process presented in Sections 5 and 6.1. However, direct connections between incoming and outgoing variables can be examined without this complex behavior model and offer an early opportunity to demonstrate an example of our analysis procedure. In this section, we examine the predictive power of the incoming parameters on performance and engagement measures summarized in Section 3.3 by following the prediction procedure described in Section 3.4.

We started our analyses by predicting both of the engagement measures (i.e., *total-actions*, and *distinct-content*) using the incoming parameters as features. Results indicated that the incoming parameters did not improve the overall fit of the model when compared to an intercept-only model when predicting both *total-actions* ($\chi_{\left(4\right)}^{2}=5.230,p=0.265$) and *distinct-content* [$\chi_{\left(4\right)}^{2}=4.146,p=0.387$]. We continued with performance prediction analyses by predicting both post-test scores and the NLG. For post-test prediction, we fitted a regression model (incoming-model) by adding rest of the incoming parameters (i.e., KMA and SE scores) as features to pre-test scores and DPA. Compared to a regression model with only pre-test scores and DPA as features, the *incoming-model* did not fit the data better [$\chi_{\left(3\right)}^{2}=0.326,p=0.955$] and none of the features related to incoming parameters were significant, except for the pre-test scores (*B* = 0.620, *t* = 5.824, *p* < 0.001) and DPA (*B* = 0.216, *t* = 2.128, *p* = 0.037). Similar to post-test prediction results, we discovered that none of the incoming parameters were significant predictors of NLG, except for marginally significant DPA feature (*B* = 0.244, *t* = 1.946, *p* = 0.056).

To sum up, in this section we observed that none of the incoming parameters were significant predictors in engagement and performance prediction, and as features, they did not improve the overall fit of the regression models.

## 5. Building Learner Practice Behavior Profile

While traditional individual differences measured in our study have no direct impact on engagement and performance (Section 4), their impact might be indirect, i.e., individual differences might influence learner practice behavior in the system and, in turn, the behavior could affect performance and engagement (Figure 2). To assess connections between incoming factors, behavior, and outcomes, we need to model learner practice behavior in some form that enables us to examine these connections formally. In this section, we present the first step of the behavior modeling procedure: building individual practice behavior profile. In the next step of the modeling procedure presented in Section 6, these profiles are used to reveal groups of users with divergent behavior and build a cluster model of practice behavior.

Given the non-mandatory nature of the practice system, students accessed practice problems and examples without predefined order or deadlines. In this “free” learning context, the order of their work with learning content is likely to be the most characteristic feature of their practice behavior. This context and past success of behavior mining approaches encouraged us to apply sequence mining for modeling practice behavior. A distinctive feature of our procedure compared to other sequence-mining approaches is representing the practice behavior of individual learners as a stable practice profile that differentiates them from each other.

### 5.1. Practice Action Labeling for Sequence Mining

The first step in sequential pattern mining is to label students' practice actions and define the specific action sequences to be mined. We believed that the sequence of interactions with learning activities and transitions between the activities (i.e., examples and problems) were critical in modeling individual differences. To pursue this idea, we performed a labeling process that highlights these critical interactions. We started the labeling process by mapping each student action to a unique label. Table 2 lists key learning actions and the corresponding labels used in the labeling process. As described earlier, practice activities were grouped into several SQL topics. To access a list of activities for a topic, a student opens a topic. Once the topic is opened, learners can work with activities of the topic in any order. With this design, student work with a topic becomes a unit of practice. To reflect this, we formed behavior sequences corresponding to learners' work with individual topics: all learning actions between two topic openings are considered to be one sequence, and each sequence starts with the topic opening label *topic-o*. We also introduced labels for opening and working with each type of content (i.e., *ex-o, ex-line*). If a student performed a content action after opening a content item (attempting a problem or viewing an explanation line), we collapsed labels for content opening and kept the labels for the actual learning actions. For example, a sequence *{prob-o, prob-o}* means that a student opened two problems consecutively without trying to solve any of them. In addition, we distinguished a failed and a successful problem solving attempt from one another to differentiate learning actions that occurred after either a failed or a successful attempt.

**Table 2:** List of labels and the corresponding learning actions that were used in the labeling process.

**Pattern label**	**Learning action**
topic-o	Opening a topic.
prob-s	Successful problem solving attempt.
ex-o	Opening an example activity.
prob-f	Failed attempt for a problem.
ex-line	Viewing an explanation line.
query-o	Opening query execution mode.
prob-o	Opening a problem.
query-e	Checking query results in query execution mode.

One of the challenges of sequence analysis of learning data is the presence of repetitive learning actions, such as a row of failed problem solving attempts, or a row of multiple line explanation views where exact number or repetition is not essential, but the relative scale of repetition is. To address it, we collapsed these sequences so that we can capture what actually happened after these repetitive actions. In this process, we first generated all sequences with repetitive labels. Then, we calculated the median length of repeated labels for all students. Then, we went over the original action sequences and replaced each repetitive label with a single uppercase version of that label if the length of that repetition was greater than the median length, or with a single lowercase label otherwise. At the end of this process, each label could represent one or more consecutive repeated actions, depending on the median length. Only *ex-line* and *query-e* had a median length of two, while others had a median length of one. As the result of the labeling process, 3432 sequences were generated from interaction logs of 88 students in the main dataset.

### 5.2. Discovering Frequent Patterns of Practice Behavior

To discover the frequent patterns in student action sequences, we used the SPAM sequence mining algorithm (Ayres et al., 2002; Fournier-Viger et al., 2016). The sequences generated after the activity labeling process were used for mining frequent patterns. To reveal sequences that could highlight individual differences, we set the minimum support for the SPAM algorithm at 0.5%. Due to our labeling process with repetition reduction, the sequences used in the mining process were already dense in information. Even if some sequences were not frequently followed (not having high levels of support), they could be important in revealing discriminative practice behaviors. The SPAM algorithm discovered 169 frequent patterns that appeared at least in 0.5% of sequences (18 sequences). All discovered patterns consist of two or three consecutive learning actions, as we did not include any gap constraint to the SPAM algorithm. Table 3 shows the top 10 most frequent patterns.

**Table 3:** Discovered top 10 frequent patterns with sequence explanations and frequency of occurrence.

**Pattern**	**Freq.(%)**	**Explanation**	**Pattern**	**Freq.(%)**	**Explanation**
{topic-o, EX-LINE}	4.8	Opening a topic followed by viewing a *long* sequence of line explanations.	{topic-o, ex-o}	2.1	Opening a topic followed by an example opening without line viewing.
{topic-o, ex-line}	2.7	Opening a topic followed by viewing a *short* sequence of line explanations.	{prob-f, prob-s}	2.0	Failed attempt followed by a successful attempt.
{topic-o, prob-o}	2.5	Opening a topic followed by a problem opening without any attempt.	{query-e, prob-s}	2.0	*Short* sequence of query executions followed by a successful attempt.
{prob-f, query-e}	2.3	Failed attempt followed by a *short* sequence of query executions.	{prob-s, prob-f}	1.8	Successful attempt followed by a failed attempt.
{topic-o, EX-O}	2.2	Opening a topic followed by a *long* sequence of example openings without line viewing.	{PROB-S, prob-f}	1.7	*Long* sequence of successful attempts followed by a failed attempt.

*Lowercase actions mean that the repetition of that action is less than or equal to the median repetition length, while uppercase actions mean the opposite*.

Out of 88 students, 82 students had at least one frequent pattern after the mining process. We further filtered out students with less than 25 frequent patterns (Q1: 45.75, Med: 97.00, M: 103.20) to have a fair amount of representation of practice behavior by the discovered frequent patterns. After the filtering process, the number of students with frequent patterns dropped to 75.

### 5.3. Building and Assessing Individual Practice Profile With Frequent Patterns

In the final step, we built a practice profile for each student as a frequency vector using the discovered 169 frequent patterns. Each position in this vector represents how many times the corresponding frequent pattern appears in the practice work of the modeled student. To eliminate any possible impact of the amount of practice, we normalized the frequency vectors per student and now the resulting vectors represent the probability of the occurrence of each frequent pattern. This approach was first introduced in Guerra et al. (2014) and successfully used to model learner behavior in Hosseini et al. (2017). Following these works, we called the practice behavior profile the *practice genome*.

To make sure that the constructed “practice genomes” in the form of probability vectors represent a sufficiently stable model of practice behavior and reliably distinguish students from each other, we checked the *stability* of the practice genomes. Following the procedure suggested in Guerra et al. (2014), we split students' sequences into two “halves” using two approaches: (1) random split, and (2) temporal split. In the random-split approach, we shuffled students' topic-level sequences and divided them into two halves randomly. In the temporal-split approach, we first ordered the sequences based on time and divided the sequences into early and late halves. For either split approach, we built separate practice “half-profiles” from each of the halves and calculated the pairwise distances for the whole set of “half-profiles” using the Jenson-Shannon (JS) divergence (as we are calculating the distance between two probability distributions). To assert genome stability, the distance between the two “half-profile” vectors of the same student (self-distance) should be smaller than the distance to half-vectors of other students (others-distance).

To evaluate this expectation, we conducted a paired *t*-test to compare the calculated *self-distances* to *others-distances* for both random-split and temporal-split approaches. The random-split self-distances (*M* = 0.35, *SD* = 0.11) were significantly smaller than the random-split other-distances (*M* = 0.46, *SD* = 0.05); *t*_(79)_ = −7.531, *p* < 0.001, *Cohen's* *d* = 0.58. Similarly, the temporal-split self-distances (*M* = 0.42, *SD* = 0.11) were significantly smaller than the temporal-split other-distances (*M* = 0.48, *SD* = 0.05); *t*_(76)_ = −5.034, *p* < 0.001, *Cohen's* *d* = 0.87. These findings showed that the practice genomes were successful in distinguishing students from each other. This property opens a way to use practice genomes for the data-driven modeling of individual differences.

## 6. Discovering Groups of Learners with Similar Practice Behavior

As reviewed in Section 2.2, the dominated approach to behavior modeling in educational data mining is discovering latent learner groups composed of students who exhibit similar behavior patterns. We follow a similar approach: we reveal these latent learner groups by clustering practice profiles of students. These clusters may be considered as a simple “cluster” model of learner behavior where individual students are modeled by their association with these latent groups. In this section, we explain the process of clustering students in groups with similar behavior and explain the process of assessing the quality of these clusters.

### 6.1. Forming Clusters of Learners Based on Practice Genomes

Given the confirmed stability, the practice genomes offer a reliable foundation to find students with similar practice behavior. The clustering of genomes was performed in two steps. First, we mapped the higher-dimensional practice genomes (i.e., 169 dimensions of the probability vectors) into a two-dimensional space by using a dimensionality-reduction technique. Next, we clustered students using the lower-dimensional representation of the practice genomes.

The main rationale behind the two-step clustering approach was that low-dimensional representation makes it easier for us to convert categorical cluster representation into a continuous behavioral scale, which is the goal of the final step of our study explained in Section 8. In our approach, we fixed the number of clusters to two (*k* = 2) by analyzing the higher-dimensional practice genomes using silhouette method (Rousseeuw, 1987) and gap statistics (Tibshirani et al., 2001).

During the first step of the clustering process, we used t-Stochastic Neighbor Embedding (t-SNE) (van der Maaten and Hinton, 2008), a non-linear dimensionality-reduction algorithm that is mainly used to explore high-dimensional data, to project practice genomes to 2-D points. t-SNE minimizes the objective function using a randomly-initiated gradient descent optimization. Thus, each run of t-SNE generates a different projection. For the results presented in this paper, we first applied a grid-search technique to tune hyper-parameters (e.g., exaggeration factor, perplexity, theta) and selected the projection that leads to the most distinct cluster separation (in Step 2), based on the frequent patterns. Thus, for the grid-search and the projection selection, we executed the first and the second step of the clustering process together for each run.

**Figure 3:**
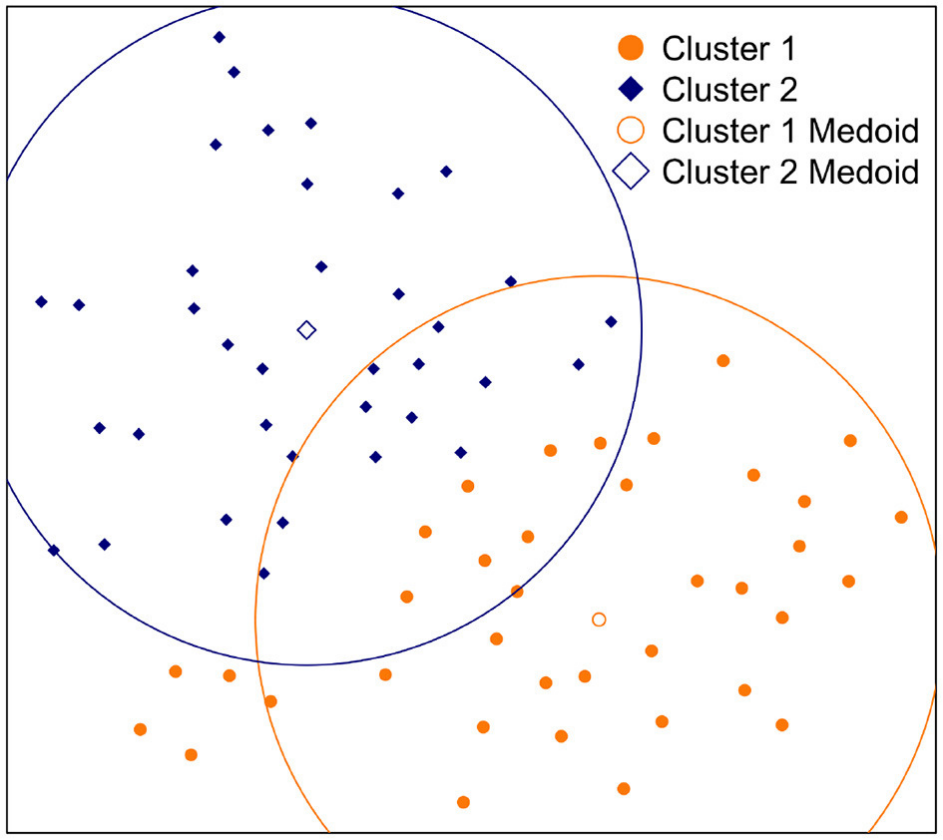
Student practice behavior representation on 2-D t-SNE projection with PAM clustering results (*k* = 2). The orange circle represents the mean Euclidean distance of all students to the Cluster 1 medoid (the orange empty square), and the blue circle represents the mean Euclidean distance of all students to the Cluster 2 medoid (the empty blue diamond).

During the second step of the clustering process, we applied partition around medoids (PAM) clustering to the 2-D results of t-SNE projections. To judge the cluster separation for the grid-search and projection selection, we performed a differential sequence mining approach similar to Kinnebrew and Biswas (2012) to compare the mean probability (ratio) of each frequent pattern between the discovered clusters (*k* = 2) using multiple *t*-tests at α = 0.05 and counted the number of significantly different patterns between each cluster. Based on this approach, we selected the 2-D t-SNE projection. The selected t-SNE projection of the practice behaviors and the PAM clustering results are presented in Figure 3. After clustering, there were 38 and 37 students in clusters 1 and 2, respectively (avg. silhouette score = 0.40).

### 6.2. Examining Cluster Separation Quality With Incoming Parameters

We need to perform quality assessment to examine whether the clustering process was successful in grouping learners with similar practice behavior. To do so, we checked the differences between clusters in various dimensions and compared the separation quality against other grouping metrics based on the incoming parameters. For all numeric metrics in the incoming parameters (e.g., pre-test, SE, KMA scores), we used median values to split students into two opposing subgroups. To examine possible impact of gender, we split students in female and male groups.

#### 6.2.1. Exploring Frequent Pattern Differences

We started our exploration by checking differences between discovered clusters in frequent patterns. We expected to find strong differences between the clusters given that practice genomes were used in clustering process. However, we performed this exploration as a sanity check and as a reference point for comparison with other grouping metrics.

We followed a differential sequence mining approach similar to Kinnebrew and Biswas (2012) to compare the mean probability (ratio) of each frequent pattern between the clusters and formed subgroups using multiple *t*-tests at α = 0.05. In this approach, there is no implemented procedure for controlling for the family-wise error rate. However, we applied Bonferroni correction to control family-wise error rate. In Table 4, we reported the total number of significantly different patterns by considering top-10, top-50 and all frequent patterns (i.e., 169) for different grouping metrics with and without performing *p*-value corrections.

**Table 4:** Summary of differential sequential mining results based on each grouping metric.

**Grouping metric**	**Top-10**	**Top-50**	**All patterns**
Cluster	5(3)	22(9)	64(7)
Pre-test	1(0)	2(0)	6(0)
KMA	0(0)	1(0)	6(0)
SE	1(0)	7(0)	22(0)
Gender	0(0)	4(0)	8(0)

*The number of significantly different patterns and (the number of significantly different patterns after Bonferroni correction) are reported by considering top-10, top-50, and all available (169) frequent patterns*.

As shown in the table, cluster separation differentiated students rigidly. When considered all 169 frequent patterns, 64 (38%) patterns found to be significantly different between clusters. For top-10 and top-50, we reached to similar conclusion where approximately half of the patterns were significantly different between clusters. More importantly, even after applying Bonferroni correction, some patterns remained significantly different that highlights the significance of pattern-based differences between clusters.

We followed the same procedure for the opposing subgroups formed based on the incoming parameters. When all 169 frequent patterns considered, the maximum number of significantly different patters was 22 (13%) for SE-based grouping. Other grouping metrics could not even reach that level of separation (see Table 4 for details). When Bonferroni correction applied^2^, none of the grouping metric had any significantly different pattern, which was not the case for clustering. However, it was interesting to observe that almost all significantly different patterns between opposing subgroups were unique to that particular grouping metric. There were 41 (25%) significantly different patterns between all opposing subgroups in total (without Bonferroni correction). Only one frequent pattern was significantly different for two grouping metric. It looks like each incoming parameter captured only limited practice behavior differences between subgroups based on frequent patterns.

To summarize, in this section, we observed that none of the collected incoming parameters was strong enough to distinguish learners as good as clusters based on frequent patterns.

#### 6.2.2. Exploring Practice Genome Differences

In the previous section, we detected only minor differences among subgroups by checking individual frequent patterns. In this section, we extended this approach and instead of checking differences in patterns, we followed a more holistic approach and explored differences between subgroups using the practice genomes. Namely, we used the same subgroups that we already formed in the previous section (including clusters) and calculated the pairwise distances between a learner's practice genome and other students' genomes in the opposing subgroups using Jenson-Shannon (JS) divergence as we used it for checking the stability of the practice genomes (see Section 5.3).

At the end of the subgroup formation, each student was assigned to a particular subgroup. For example, a male student might belong to Cluster 1, high SE, low pre-test, and low KMA subgroups. Thus, for this particular student, we can calculate the average distance of his practice genome to the genomes of all students in the opposing subgroups; i.e., female group, Cluster 2, low SE, high pre-test, and high KMA subgroups. At the end of this process, each student had five average genome distances to the opposing subgroups. In total, we calculated 70 average genome distances for each grouping metric, one for each student.

If one of the grouping metric was more distinguishing than the others, we expect that the average distance to that opposing subgroup should be the maximum compared to others. To check this hypothesis, we performed multiple paired *t*-tests to compare average pairwise distance values for each subgroup and further adjusted *p*-values using Bonferroni correction. Results showed that the cluster based grouping generated significantly higher pairwise JS distance compared to other four grouping metrics [Pre-test: *t*_(69)_ = 6.290, *p* < 0.001; KMA: *t*_(69)_ = 7.921, *p* < 0.001; SE: *t*_(69)_ = 5.821, *p* < 0.001; Gender: *t*_(69)_ = 7.773, *p* < 0.001]. Similar to pattern based exploration performed in the previous section, we conclude that clustering was the most powerful grouping metric to separate learners' practice genomes compared to other groupings created by the incoming parameters.

### 6.3. Practice Behavior Differences Between Clusters

**Figure 4:**
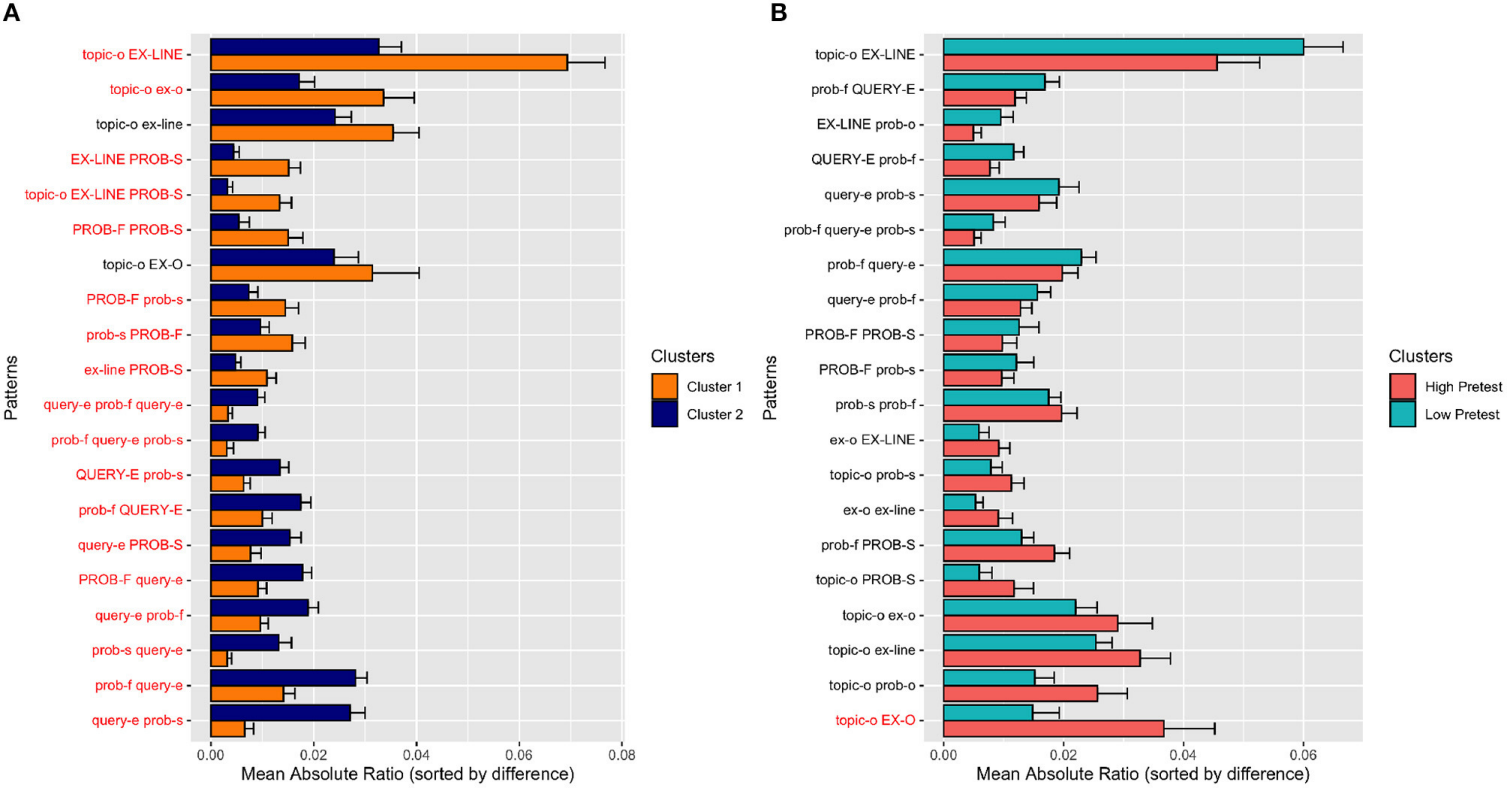
Frequent pattern comparison between **(A)** discovered clusters and **(B)** high/low pre-test students. The frequent patterns are selected to highlight the highest absolute ratio difference between two groups, and sorted by mean ratio (probability) difference. Red texts denote significantly different patterns between two clusters, based on the results of the *t*-test without applying any *p*-value correction.

By examining the cluster separation quality in the previous section, we quantified differences between clusters based on individual frequent patterns and practice genomes; and compared to other subgroups based on each incoming parameter. The eventual goal of the clustering process was to discover latent student groups with considerably different practice behavior. Thus, we continue assessing the quality of clustering process by analyzing practice behavior differences between clusters. To achieve this, we calculated the average ratio (probability) of frequent patterns in both clusters. In Figure 4A, we plotted the average ratio of 20 patterns that had the highest absolute ratio difference between two clusters and sorted them by the difference of the absolute ratio. In the figure, there are 10 patterns that more frequently occurred in Cluster 1 (top half of the graph) and 10 patterns that more frequently occurred in Cluster 2 (bottom half of the graph). The significantly different patterns are labeled with a red colored text.

As shown in the figure, students in two clusters exhibited considerably different practice behavior. Students in Cluster 1 significantly more frequently opened and explored examples right after they began to work with a topic (e.g., {topic-o, EX-LINE}, {topic-o, ex-o}). Moreover, they switched more frequently from viewing explanations to successful problem solving, suggesting that they valued examples as a preparation tool for problem solving (e.g., {EX-LINE, PROB-S}, {topic-o, EX-LINE, PROB-S}). The students in this cluster were also engaged significantly more frequently in a sequence of uninterrupted problem solving attempts, in which a sequence of failed attempts was followed by a sequence of successful attempts (e.g., {PROB-F, PROB-S}, {PROB-F, prob-s}).

In contrast, students in Cluster 2 interleaved attempts to solve problems by using the query execution mode. As seen in the figure, all 10 “distinguishing” patterns that involved the query execution mode (e.g., query-e) were significantly more frequent in Cluster 2. For example, when Cluster 2 students failed on a problem, they checked their query results in the query execution mode to get more detailed feedback (e.g., {prob-f, query-e}, {PROB-F,query-e}). Further, they typically managed to solve problems after using the query execution mode (e.g., {query-e, prob-s}, {query-e, PROB-S}, {prob-f, query-e, prob-s}. In some cases, they used the query execution mode even after successfully solving a problem (e.g., {prob-s, query-e}), suggesting that at particular cases they wanted to verify their correct queries by checking the actual query result.

To provide a side-by-side comparison, in Figure 4B, we demonstrated the average ratio (probability) of 20 frequent patterns that had the highest absolute difference between low and high pre-test students. Based on the figure, we can speculate that students with high prior knowledge exhibited more skimming behavior (e.g., {topic-o, EX-O}, {topic-o, prob-o}, {topic-o, ex-o}). However, given the only significantly different pattern, {topic-o, EX-O}, it was hard to strongly support this hypothesis. Compared to discovered clusters, it can be easily noticed that pre-test based grouping was not highly successful in separating students according to the frequent patterns even if we concentrated on the most divergent patterns between opposing subgroups.

In summary, by clustering practice genomes, we discovered two divergent practice behaviors. To simplify the difference, Cluster 1 students tended to learn by distilling SQL knowledge encapsulated in examples and then applying it to practice problems. Cluster 2 students preferred to “generate” SQL knowledge through their own experience obtained by experimenting with various SQL queries, which they used as exploration, debugging, and verification tools.

## 7. Connecting Practice Behavior to Incoming and Outgoing Parameters through Cluster Model

The clusters of learners obtained through the profiling and clustering process explained above represent a simple formal model of learner behavior, which is currently typical for data-driven studies of learner behavior in the field of educational datamining (see Section 2.2). With this cluster model, individual learners are modeled by their association with one of the behavior clusters. The ability to compress complex learner behavior into a simple model enables us to investigate two groups of connections displayed in Figure 2 that we were not able to assess before. The next (Section 7.1) explores the connections between incoming parameters and learning behavior modeled by clusters. Following that, Section 7.2 investigates the connections between the clusters and the outgoing parameters (engagement and performance).

### 7.1. Connecting Clusters to Incoming Parameters

Since the discovered clusters revealed some divergent learning behavior, it is important to check whether the observed behavioral differences could be explained by some incoming parameters collected in our study (i.e., gender, pre-test, SE, and KMA scores). To draw the connection from the obtained clusters to incoming parameters, we checked whether there are any other noticeable differences between behavior clusters in terms of each of these parameters. We summarized the incoming parameters for each cluster in Table 5. For each incoming parameter, we only considered students with available data. As seen in the table, the clusters were balanced in respect to each of the incoming parameters. The percentage of female students were 56 and 44%, respectively. The mean scores of SE, KMA, and pre-test were also very similar. Our formal analysis did not reveal any statistically significant differences in SE scores [*t*_(68)_ = −0.53, *p* = 0.60], KMA scores (*U* = 468, *p* = 0.43), and pre-test scores (*U* = 645, *p* = 0.52) between the behavior clusters.

**Table 5:** Summary of the incoming parameters, engagement and performance measures for the discovered clusters.

		**Incoming parameters**				**Engagement**		**Performance**	
		**Female**	**SE**	**KMA**	**Pre-test**	**Total-**	**Distinct-**	**Post-test**	
**Clusters**	**N**	**%**	**score**	**score**	**score**	**actions**	**content**	**score**	**NLG**
Cluster 1	38	56	21.2(4.2)	0.53(0.44)	1.2(1.5)	485(258)	233(80)	5.2(1.6)	0.45(0.14)
Cluster 2	37	44	21.7(4.4)	0.60(.45)	1.4(1.6)	600(304)	226(87)	4.8(1.9)	0.39(0.20)

*Mean and (SD) are reported*.

To draw the connection in the opposite direction, from the incoming parameters to the clusters, we fitted a binomial generalized linear model to predict categorical cluster labels using pre-test, KMA, SE scores, and gender. Compared to an intercept-only model, the incoming parameters did not improve the overall fit of the model [$\chi_{\left(3\right)}^{2}=1.383,p=0.710$] and achieved a very low area under the ROC curve (AUC) of 0.587, which suggests that cluster assignments cannot be explained by the incoming parameters.

The lack of connections between traditional dimensions of individual differences and other incoming parameters collected in our study and learner practice behavior indicates that observed differences in practice behavior may reflect a latent dimension of individual differences that can't be reduced to traditional measures, but could be revealed through divergent learner behavior. This observation enables us to treat the cluster behavior model as a *simple* data-driven model of individual differences.

### 7.2. Connecting Clusters to Outgoing Parameters

In this section, we attempt to connect the cluster model of practice behavior to outgoing parameters (i.e., engagement and performance measures as described in Section 3.3). Similarly to the previous subsection, we perform this analysis in two different ways. We first checked if there were any observable differences between clusters for each engagement and performance measures and we only considered students with available data for each measure. Table 5 reports the summary of the incoming and outgoing parameters for each cluster. We found out that there were no statistically significant differences between clusters in *total-actions* (*U* = 539, *p* = 0.08), *distinct-content* (*U* = 739, *p* = 0.71), post-test scores [*t*_(70)_ = 0.88, *p* = 0.38], and NLG (*U* = 733, *p* = 0.34). In other words, the clusters can't be directly explained by differences in the outgoing parameters (what we see are not clusters of “good learners” and “bad learners”, neither they are clusters of “well-enageged” and “poorly-engaged” learners).

We further fitted regression models to predict outgoing parameters. This analysis could be also considered as an attempt to assess the predictive power of our simple data-driven model of individual differences. We added the categorical cluster labels as a predictor in these fitted models and followed the prediction procedure described in Section 3.4. Table 6 summarizes the fitted regression models. Results indicated that the model that used categorical cluster labels (cluster-model) to predict *total-actions* did not improve the overall fit of the model as compared to an *intercept-only* model [$\chi_{\left(1\right)}^{2}=2.55,p=0.110$] and the *incoming-model* (i.e., model that used incoming parameters as features that was fitted in Section 4) [$\chi_{\left(3\right)}^{2}=2.677,p=0.444$]. Similarly, the *cluster-model* did not fit the data better in *distinct-content* prediction when compared to the *intercept-only* [$\chi_{\left(1\right)}^{2}=0.018,p=0.893$] and *incoming-model* [$\chi_{\left(3\right)}^{2}=4.128,p=0.248$]. In post-test scores prediction, cluster labels did not improve the overall fit of the model compared to the *intercept-only* [$\chi_{\left(1\right)}^{2}=1.133,p=0.287$] and *incoming-model* [$\chi_{\left(2\right)}^{2}=0.807,p=0.668$]. However, compared to the *incoming-model*, the *cluster-model* improved the explained variance by 2.7% (based on adj. *R*^2^). In parallel, for NLG prediction, the *cluster-model* did not predict NLG better compared to both *intercept-only* [$\chi_{\left(1\right)}^{2}=0.975,p=0.324$] and *incoming-model* [$\chi_{\left(1\right)}^{2}=0.757,p=0.685$]. Yet, it improved the explained variance by 2.1%. This result indicates that the cluster model treated as a simple data-driven model of individual differences could be more useful for predicting important outgoing parameters of learning than traditional models. This finding motivated the last stage of our work presented in the next section.

**Table 6:** Summary of the fitted regression models to predict engagement and performance measures by categorical cluster labels.

	**Dependent variable:**			

	***Total-actions***	***Distinct-content***	***post-test score***	***NLG***
	***negative***	***negative***	***OLS***	***OLS***
	***binomial***	***binomial***		
Pre-test score			0.639^***^	
DPA			0.243^**^	0.275^**^
Cluster (C2)	0.214	–0.014	–0.203	–0.241
Adjusted R^2^			0.408	0.045
Log Likelihood	–491.666	–417.967		
Akaike Inf. Crit.	987.331	839.934		
F Statistic			15.919^***^	2.514
			(df = 3; 62)	(df = 2; 63)

*Standardized regression coefficients are reported for the independent variables.*

** *p < 0.05;*

*** *p < 0.01*.

## 8. Developing a Data-Driven Model of Individual Differences

This section focuses on the final goal of our study: building a data-driven model of individual differences that could complement existing dimensions of individual differences and improve our ability to connect learner practice behavior to performance and engagement. In the previous section, we demonstrated the presence of two clusters that exhibit significantly divergent practice behavior. We also observed that these behavior differences can't be explained by incoming parameters hinting that the observed divergent behavior could be used to construct a data-driven model of individual differences, which is “orthogonal” to the traditional models of individual differences collected in our study. While our simple data-driven model of individual differences was not able to offer significant help in prediction tasks, our attempts hinted that it could be more useful than traditional models (Section 7.2).

However, the comparison of the *simple* data-driven model with the traditional models was not fully fair. In contrast to traditional models that usually offer a continuous scale between two opposite ends (i.e., low/high self-esteem, etc), our simple “binary” model has to fully allocate every student to one of the latent groups, which might be too simplistic to offer a good modeling and predictive power. In this section we attempt to refine our data-driven binary model into a more traditional continuous behavioral scale and evaluate the predictive power of the data-driven scale on various engagement and performance measures (see Section 3.3 for details about measures). We compare the relative predictive power of the behavioral scale (i.e., distance to cluster medoids) against the incoming parameters (i.e., gender, pre-test scores, KMA, and SE scores) and the categorical behavioral cluster representations. Further, we check the transferability of the constructed behavioral scale using the *transfer* dataset.

### 8.1. Developing a Continuous Behavioral Scale

In this section, we attempted to refine the categorical cluster assignments into a continuous behavioral scale that can model individual differences reflected through the practice behavior, similar to traditional scales of individual differences.

To follow existing “bi-polar” scales of individual differences, we attempted to quantify the position of a student with respect to each main practice behavior (depicted by the clusters) as the Euclidean distance from the student's 2-D point to the cluster medoids found by the PAM clustering algorithm. Thus, we modeled the practice behavior of a student using two numerical values: (1) distance to the first cluster medoid (M1), and (2) distance to the second cluster medoid (M2).

**Figure 5:**
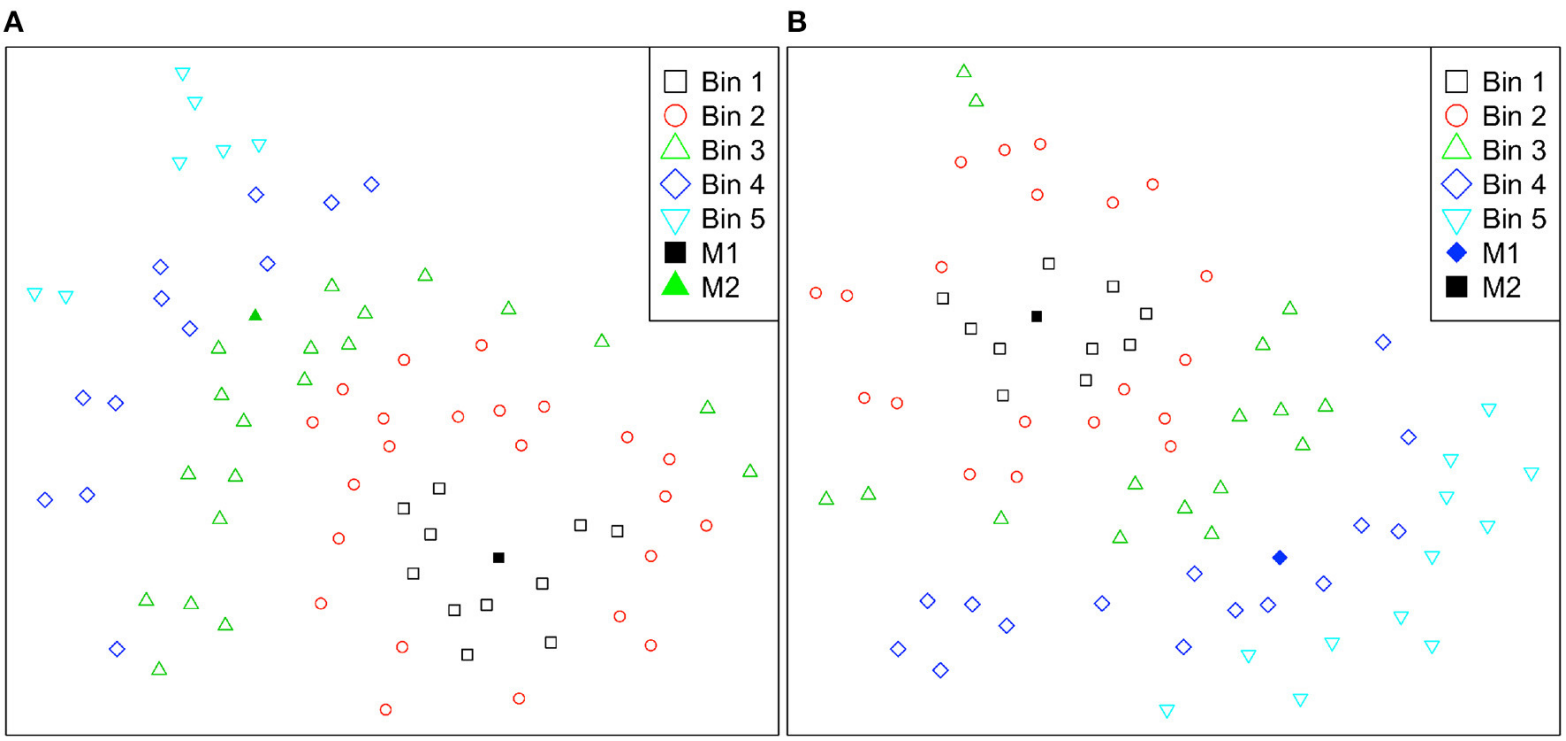
Student practice behavior representations are divided into five bins according to their Euclidean distances to cluster medoids and color-coded for easier interpretation on the 2-D t-SNE projection. Bins are numbered from 1 to 5 in increasing average distance for **(A)** Cluster 1 medoid (M1) and **(B)** Cluster 2 medoid (M2).

To investigate how distances to cluster medoids captured differences among students (i.e., incoming parameters, engagement, and performance), we divided students into five *bins* using Euclidean distances on 2D dimension. The bins are numbered from 1 to 5 in increasing average distance for M1 and M2, where bin 1 is the closest group to the medoids, as illustrated in Figure 5. As Table 7 shows, grouping based on distance to M1, the average number of distinct explanation lines viewed drops considerably as the distance increases, and we found a significant negative correlation (*r* = −0.48, *p* < 0.001). We also found a weak positive correlation with the average number of query executions (*r* = 0.23, *p* = 0.04), but there was no constant decrease or increase based on the distance. Thus, with the increase of the distance to M1, the number of distinct line views decreases and the number of query executions increases. For grouping based on the distance to M2, we found a significant negative correlation between distance and the number of distinct problems attempted (*r* = −0.30, *p* = 0.009), and between distance and the number of query executions (*r* = −68, *p* < 0.001). We also found a significant positive correlation with the NLG (*r* = 0.24, *p* = 0.038). Thus, we can summarize that when students move away from M2, the NLG increases while they attempted fewer distinct problems and performed fewer number of query executions. The correlations summarized in this section overlaps with the practice behaviors described in Section 6.3, where students in Cluster 1 were more concentrated on examples and students in Cluster 2 were performing more query executions.

**Table 7:** Summary of groups obtained by dividing students into five bins based on the Euclidean distances to the two cluster medoids on 2D dimension.

		**Incoming parameters**				**Performance**		**Practice system usage**			
		**Female**	**SE**	**KMA**	**Pre-test**	**Post-test**		**Problem**	**Example**	**Line**	**Query**
	**N**	**%**	**score**	**score**	**score**	**score**	**NLG**	**attempts**	**views**	**views**	**executions**
**Distance to**											
**Medoid 1**											
Bin 1	12	40	21.80	0.46	1.17	5.40	0.49	41.50	60.50	170.00	51.25
Bin 2	23	55	21.65	0.57	1.37	4.96	0.41	37.00	56.13	152.39	35.30
Bin 3	21	56	21.68	0.65	1.24	4.78	0.41	35.76	56.47	141.86	90.14
Bin 4	12	42	20.17	0.50	1.17	4.83	0.41	41.08	56.00	93.75	81.92
Bin 5	7	50	21.67	0.59	1.71	5.17	0.39	35.28	52.43	61.57	61.57
**Distance to**											
**Medoid 2**											
Bin 1	11	44	22.27	0.59	0.45	4.27	0.40	42.18	58.73	156.72	153.36
Bin 2	20	53	21.89	0.59	1.82	4.73	0.34	42.35	57.55	124.80	86.65
Bin 3	16	43	19.93	0.51	0.81	5.00	0.46	36.81	55.19	123.81	38.31
Bin 4	16	43	21.00	0.63	2.06	5.80	0.48	34.63	56.31	125.13	31.50
Bin 5	12	70	21.17	0.49	0.83	4.96	0.45	32.17	55.08	156.42	16.42

*Mean values are reported*.

To check if there was any connection between the distance values and the incoming parameters, we fitted linear regression models to predict both distance values by adding pre-test, KMA, SE scores and gender as predictors. We did not find any significant correlation between distance and the incoming parameters. This means that we cannot explain distances to cluster medoids and the attributed practice behavior by the incoming parameters.

### 8.2. Predicting Engagement With Behavioral Scale

We began our analyses by predicting *total-actions* with the behavioral scale, i.e., distances to cluster medoids. Regression results indicated that the model with distance to medoids as features (distance-model) fitted data significantly better than both the intercept-only model [$\chi_{\left(2\right)}^{2}=14.744,p<0.001$] and the model that used the incoming parameters (incoming-model) as features [$\chi_{\left(2\right)}^{2}=9.514,p=0.009$]. Moreover, the *distance-model* also fitted data significantly better than the model that used categorical cluster labels (cluster-model) [$\chi_{\left(1\right)}^{2}=12.191,p=0.001$]. We further fitted a separate model with both distance measures and individual parameters together as features (full-model) to compare relative regression coefficients. In this model, distance to M1 and distance to M2 were the only significant predictors of *total-actions*, where moving away from both medoids was associated with a fewer number of actions. We can mention that the distance to M2 had a slightly higher negative effect on *total-actions*.

We continued predicting engagement measures with *distinct-content*. Comparable to the results for *total-actions* prediction, we found out that the *distance-model* fitted the data significantly better than the *intercept-only* model [$\chi_{\left(2\right)}^{2}=10.575,p=0.005$], the *cluster-model* [$\chi_{\left(1\right)}^{2}=10.557,p=0.001$], and the *incoming-model* [$\chi_{\left(2\right)}^{2}=6.428,p=0.004$]. Next, we fitted a model with both distance and incoming parameters together (full-model) and found that only the distance measures were significant predictors. Based on the regression coefficients, we saw that the distance to M1 had a higher negative impact on *distinct-content* compared to the distance to M2. Table 8 presents the summary of the fitted models for predicting engagement measures.

**Table 8:** Summary of the fitted regression models to predict engagement and performance measures.

***Dependent variables***	***total-actions***		***distinct-content***		***Post-test score***		***NLG***	
***Regression Type***	***Negative binomial***		***Negative binomial***		***OLS***		***OLS***	
***Model Name***	***Distance-model***	***Full-model***	***distance-model***	***Full-model***	***Distance-model***	***Full-model***	***Distance-model***	***Full-model***
Pre-test score		–0.002		0.0001	0.611^***^	0.587^***^		
Gender (M)		0.124		0.050		0.112		0.258
SE score		0.097		0.071		–0.031		–0.075
KMA score		–0.071		–0.053		–0.012		–0.019
DPA					0.293^***^	0.291^***^	0.354^***^	0.353^***^
Dist. to M1	–0.216^***^	–0.207^***^	–0.201^***^	–0.196^***^	0.124	0.124	0.194	0.184
Dist. to M2	–0.292^***^	–0.289^***^	–0.121^**^	–0.121^**^	0.264^**^	0.266^**^	0.365^**^	0.361^**^
Adjusted R^2^					.435	0.409	0.104	.083
Log likelihood	–485.570	–483.257	-412.689	–410.900				
Akaike Inf. Crit.	977.141	980.513	831.378	835.801				
F Statistic					13.486^***^	7.434^***^	3.527^**^	1.984
					(df = 4; 61)	(df = 7; 58)	(df = 3; 62)	(df = 6; 59)

*Standardized regression coefficients are reported for the independent variables.*

** *p < 0.05;*

*** *p < 0.01*.

In summary, students who were close to M2 performed more learning actions, which can be explained by the overall practice behavior of Cluster 2: they failed more on problem attempts and used query execution more frequently, as compared to Cluster 1. Students who were close to M1 covered more unique content, such as viewing more unique explanation lines.

### 8.3. Predicting Performance With Behavioral Scale

In this section, we advanced to performance prediction by predicting both post-test scores and the NLG by using the behavioral scale. The summary of the fitted models is presented in Table 8.

To predict post-test scores, we fitted a regression model with distance to medoids as features (distance-model). We found out that the *distance-model* fitted the data significantly better than the *incoming-model* (i.e., the model with incoming parameters as features) [$\chi_{\left(1\right)}^{2}=4.928,p=0.026$], and better than the model that used binary cluster assignments, pre-test scores and DPA as features [$\chi_{\left(1\right)}^{2}=4.121,p=0.042$]. Next, we fitted a model with all features together (full-model), and these results indicated that after pre-test scores and DPA, the distance to M2 was the only significant predictor. Thus, after controlling for the prior knowledge and the number of distinct problems attempted, the distance to the second cluster medoid significantly predicts post-test scores.

In NLG prediction, we discovered that the distance to M2 significantly predicted NLG after controlling for the DPA in the *distance-model*. We further fitted a model with all features together (full-model), and again, only DPA and distance to M2 were significant predictors. Given the positive sign of the M2 regression coefficients in both post-test and NLG predictions, we concluded that the distance from the Cluster 2 medoid was associated with higher performance.

### 8.4. Transferability of Performance Prediction

In this section, we assess the transferability of our data-driven modeling approach by predicting the performance of students in a new dataset (i.e., transfer dataset) that was not used in discovering the clusters and building the behavior scale. In addition, to assess whether the data-driven modeling approach can be used for early prediction of student performance, we only used students' action sequences from the first half of the course. There were 36 students who used the practice system (attempted at least one problem and viewed at least one example) in the *transfer* dataset. We filtered out students who did not take both pre- and post-tests and who did not have any frequent patterns that could be used to build the practice genome. After this filtering process, 27 students remained.

The main challenge in this process was projecting new students' practice genomes on an already constructed 2-D tSNE projection, as shown in Figure 3, because the t-SNE algorithm learns a non-parametric mapping. To overcome this challenge, we trained a multivariate regression model to predict the location (x and y coordinates) of new practice genomes on a 2-D map using the practice genomes from the *main* dataset. This way, we can predict new students' locations and calculate their distances to the same cluster medoids.

Using the first-half sequences, we discovered 109 frequent patterns following the same approach presented in Section 5, where 102 of the discovered patterns overlapped with the previously discovered patterns (i.e., 169 frequent patterns) and 49 of them overlapped with the previously discovered top-50 frequent patterns from the *main* dataset. To build the model without overfitting, we only used these overlapping frequent patterns as features and further reduced this set to 28 patterns by applying a multivariate backward step-wise feature selection procedure. The final trained model explained the variance in the coordinates reasonably well (x: *adj*.*R*^2^ = 0.89, y: *adj*.*R*^2^ = 0.83) and convinced us to proceed. Using this model, we predicted the locations of new students on the 2-D map and calculated Euclidean distances to both medoids.

Similar to the analyses in Section 8.3 to predict post-test scores, in addition to the DPA feature, we added pre-test scores to control for levels of prior knowledge. Since we did not have incoming parameters in the *transfer* dataset, we can only report prediction results of the distance measures on this dataset. Our results indicated that the overall model was significant [$F_{\left(4,\#22\right)}=5.365,p=0.004,adj.R^{2}=0.40$]. Compared to the same model fitted in the *main* dataset, we lost 3.4% in explained variance (based on adj. *R*^2^, 0.435 in *main* dataset and 0.401 in *transfer* dataset), but this finding could simply be a result of using only the half-sequences of students. Based on the regression results, similar to our previous findings, the distance to M2 was a significant predictor(*B* = 0.642, *t* = 2.588, *p* = 0.017) but not the distance to M1 (*B* = −0.357, *t* = −1.312, *p* = 0.203). In NLG prediction, we found a similar trend where the distance to M2 was a significant feature (*B* = 0.605, *t* = 2.072, *p* = 0.049) but not the distance to M1 (*B* = −0.525, *t* = −1.758, *p* = 0.092). These results indicate that the data-driven model of practice behavior that was built by using the *main* dataset represents a reasonably stable dimension of individual differences that could be used in new datasets to predict learner performance.

## 9. Discussions and Conclusion

In this paper, we examined connections between several traditional dimensions of individual differences, practice behavior, engagement and performance in an online practice system for learning SQL. As a part of this process, we used sequence mining approach to build profiles of learner practice behavior as a probability vector of frequent patterns (i.e., practice genomes) and discovered clusters of learners with significantly divergent behavior. We discovered that none of the incoming parameters was useful to predict learner practice behavior. We considered these results as an indication that differences in learner practice behavior reflected a new latent dimension of individual differences that can not be reduced to other dimensions modeled in the study. Our data also demonstrated that even a simple cluster-based model of learner behavior was more useful in predicting engagement and performance than the established scales of individual differences such as KMA and SE. On the final step of our study, we attempted to convert the simple cluster model of practice behavior into a complete data-driven model of individual differences using a continuous behavioral scale. We evaluated this scale against the *main* dataset and examined the transferability of our modeling approach against a new semester-long dataset. Our findings showed that the data-driven behavioral scale can predict both learners' engagement within the online practice system and their performance. This results demonstrated that our data-driven model not only offers a much stronger connection to practice behavior than traditional models of individual differences, but is also more useful than traditional models in predicting engagement and performance.

An interesting “side” result that we observed is that “closeness” of practice profile to one of the cluster medoids was associated with higher post-test scores and NLG. However, we obtained this result after controlling for the practice “efforts.” This finding indicates that learner performance is not defined solely by the sheer amount of practice efforts, it is also important how a student practiced.

We believe that our results offer valuable contributions to the study of individual differences in education, the role of AI, and data analytic. As the results show, the data-driven individual differences might be better in predicting both the engagement and the performance than traditional individual differences. These results indicate the critical power of learner data in the studies of individual differences. One important finding is that modern learning environments offer students an unusual level of freedom in choosing what, when, and how to learn. With this freedom of choice, students might have higher chances to expose latent individual differences through their practice behavior, which enables researchers to collect valuable student data to discover new dimensions of individual differences through data-driven approaches. Compared to traditional models that are formed through relatively brief subjective questionnaires, the data-driven models that leverage a large volume of learner data could be both more reliable and sophisticated. From the prospect of AI, it is critical to confirm that modern AI-based data mining technologies might successfully uncover latent individual differences captured in learner data. While our study demonstrated the value of our specific approach based on sequence mining, dimensionality reduction, and clustering, we believe that researchers could obtain more impressive results with a range of methods reviewed in Section 2. In turn, a higher predictive power of data-driven individual differences opens a range of opportunities for building AI-based interventions that can make the learning process more productive and successful.

The reported results are interesting and important, but our study does have limitations. The first group of limitations is related to the measures applied. Based on multiple regression analysis, we showed that traditionally modeled individual differences such as SE and KMA were not effective in both engagement and performance prediction. This finding supports the prior research showing that SE has no impact on performance (Baumeister et al., 2003). However, the SE measure used in this study focuses on global self-worth. More specific *self-concept* constructs had a stronger relationship to the academic achievement (Marsh and Craven, 2006). In addition, we administered KMA on SQL problems that required students to write short SQL statements without having any options to select. We believe that the nature of such problems might reduce the predictive power of KMA. Other modified versions of similar measures could be used (Gama, 2004) or knowledge assessment could be monitored during usage of the practice system. In addition, the collected individual differences were limited and researchers should consider collecting other possible measures to model learners' individual differences. Finally, the performance measures that we used in this paper were based on pre/post tests rather than on actual course grades, due to having no access to this information.

Since the practice was system offered as a non-mandatory resource, our analyses are subject to self-selection bias, where we can only observe the practice behavior of students who decided to use the system. The design of the practice system adds another limitation to our findings, where students had freedom to choose topics and content on which to work freely. In addition, we collected the data from similar student cohorts attending the same graduate-level course at a large North American university. The results presented in this paper might not be transferable to other cohorts or cultures. Similar studies and analyses should be conducted in other courses and in other cultures in different settings to assess the generality of the study results. Finally, in our results, we are not claiming any causality. In our future work, we plan to apply this modeling approach to a newer version of the practice system that has more types of learning content, explore cultural differences, incorporate other self-reported measures, and check the differences among practice behaviors in the presence of different engagement manipulations.

## Data Availability Statement

The datasets presented in this article are not readily available because data protection regulations. Requests to access the datasets should be directed to kaa108@pitt.edu.

## Ethics Statement

The studies involving human participants were reviewed and approved by the Human Research Protection Office (HRPO), University of Pittsburgh. The participants provided their written informed consent to participate in this study.

## Author Contributions

KA lead the data analysis and the process of manuscript writing. PB lead the project, provided feedback, and contributed to manuscript writing. Both authors contributed ideas for study and contributed to the manuscript.

## Funding

This material is based upon work supported by the National Science Foundation under grant no. 1740775.

## Conflict of Interest

The authors declare that the research was conducted in the absence of any commercial or financial relationships that could be construed as a potential conflict of interest.

## Publisher's Note

All claims expressed in this article are solely those of the authors and do not necessarily represent those of their affiliated organizations, or those of the publisher, the editors and the reviewers. Any product that may be evaluated in this article, or claim that may be made by its manufacturer, is not guaranteed or endorsed by the publisher.

## Footnotes

^1^SE&KMA (*r=0.14, p = 0.26*); SE&pre-test (*r=0.08, p = 0.26*); SE&post-test (*r = 0.00, p = 0.98*); SE&NLG (*r=–0.13, p = 0.27*); KMA&pre-test (*r = –0.15, p = 0.24*); KMA&post-test (*r = –0.12, p = 0.34*); KMA&NLG (*r = –0.05, p = 0.72*).

^2^We reached exactly the same results when applied less conservative *p*-value adjustment procedures such as false discovery rate (FDR), and Holm-Bonferroni.
